# Triplebody Mediates Increased Anti-Leukemic Reactivity of IL-2 Activated Donor Natural Killer (NK) Cells and Impairs Viability of Their CD33-Expressing NK Subset

**DOI:** 10.3389/fimmu.2017.01100

**Published:** 2017-09-08

**Authors:** Stephan Kloess, Alessa Ede Valverde da Silva, Olaf Oberschmidt, Tanja Gardlowski, Nadine Matthies, Maulik Vyas, Lubomir Arseniev, Michael Heuser, Elke Pogge von Strandmann, Ulrike Köhl

**Affiliations:** ^1^Institute for Cellular Therapeutics, IFB-Tx, Hannover Medical School (MHH), Hannover, Germany; ^2^Department I of Internal Medicine, University Hospital of Cologne, Cologne, Germany; ^3^Department of Hematology, Hemostasis, Oncology, and Stem Cell Transplantation, Hannover Medical School (MHH), Hannover, Germany; ^4^Experimental Tumor Research, Center for Tumor Biology and Immunology, Philipps University Marburg, Marburg, Germany

**Keywords:** natural killer cells, natural killer group 2 member D, triplebodies, ULBP2, CD19, CD33, immunoligands, acute myeloid leukemia

## Abstract

Natural killer cells (NK) are essential for the elimination of resistant acute myeloid and acute lymphoblastic leukemia (AML and ALL) cells. NK cell-based immunotherapies have already successfully entered for clinical trials, but limitations due to immune escape mechanisms were identified. Therefore, we extended our established NK cell protocol by integration of the previously investigated powerful trispecific immunoligand ULBP2-aCD19-aCD33 [the so-called triplebodies (TBs)] to improve the anti-leukemic specificity of activated NK cells. IL-2-driven expansion led to strongly elevated natural killer group 2 member D (NKG2D) expressions on donor NK cells which promote the binding to ULBP2^+^ TBs. Similarly, CD33 expression on these NK cells could be detected. Dual-specific targeting and elimination were investigated against the B-cell precursor leukemia cell line BV-173 and patient blasts, which were positive for myeloid marker CD33 and B lymphoid marker CD19 exclusively presented on biphenotypic B/myeloid leukemia’s. Cytotoxicity assays demonstrated improved killing properties of NK cells pre-coated with TBs compared to untreated controls. Specific NKG2D blocking on those NK cells in response to TBs diminished this killing activity. On the contrary, the observed upregulation of surface CD33 on about 28.0% of the NK cells decreased their viability in response to TBs during cytotoxic interaction of effector and target cells. Similar side effects were also detected against CD33^+^ T- and CD19^+^ B-cells. Very preliminary proof of principle results showed promising effects using NK cells and TBs against primary leukemic cells. In summary, we demonstrated a promising strategy for redirecting primary human NK cells in response to TBs against leukemia, which may lead to a future progress in NK cell-based immunotherapies.

## Introduction

Natural killer (NK) cells are a subset of lymphoid effector cells within the innate immune response and have been shown to be a suitable tool for adoptive immunotherapy because of their ability of anti-tumor surveillance (1–6). In contrast to T cells, NK cells identify and eliminate malignant and virus-infected target cells in a major histocompatibility complex (MHC)-unrestricted way by engaging natural cytotoxicity receptors (NCRs), such as NKp30, NKp44, NKp46, and the activating receptor NKG2D (natural killer group 2 member D) which recognizes a variety of well-defined ligands expressed by transformed cells (7–9).

Major histocompatibility complex class I-related chain A and B (MICA/B) and the UL-16 binding protein family are cancer cell surface ligands that interact with NKG2D on NK cells. These specific bindings between NKG2D and their corresponding ligands (NKG2DL) on cancer cells are responsible for improved cytotoxic properties of NK cells against tumor and leukemia cells (10–12). Based on these receptor–ligand bindings between effector and target cells, an increased secretion of granzyme A (GraA) and B (GraB), granulysin, and perforin induced in NK cells could be demonstrated (13–15).

However, several types of cancer have developed a broad spectrum of immune escape mechanisms that down-modulate the NKG2D-mediated immune surveillance by metalloproteinases-driven proteolytic shedding and release of soluble NKG2DLs (16–20). In addition, elevated DNA-“hyper”-methylations for NKG2DLs could be detected in some malignant cells, mainly in acute myeloid leukemia (AML) cells, resulted in a clearance of NKG2DL surface cell expression, also detected for MICA, ULBP1/2 in AML patients (21–23). Enhanced tumor-shedding and DNA-methylation could contribute to an unhampered proliferation and evasion of immune control in AML patients (21).

Previous reports indicated that human leukocyte antigen (HLA) class I diversities could be responsible for induction of NK cell alloreactions by KIR (killer-cell immunoglobulin-like receptors)-ligand mismatch as shown in acute leukemia patients. The efficacy of this donor NK cell alloreactivity in mismatched hematopoietic transplants resulted in strong graft-versus-leukemia effects, prevented graft rejection and graft versus host disease and protected against AML relapse (24–26). Increased eliminations of AML blasts could be also shown by adoptive transfer of haploidentical NK cells and IL-2 infusions to stimulate *ex vivo* donor NK cell expansion. However, limitations have been observed by lacking of antigen specificity and long-lasting increase of immunosuppressive regulatory T cells that resulted in a reduction of NK cell proliferations and/or cytotoxic properties (27–30).

Some of the current anti-leukemia therapy studies focus on developing antibody constructs that target activated NK cells to specific leukemia antigens to overcome those limitations listed here on the functionality, expansion, and persistence of NK cells. Recent advances were made, including manipulation of receptor-mediated activation, augmentation of antibody-dependent cellular cytotoxicity reactions, gene-modified NK cells engineered by chimeric antigen receptors or, finally, mono-, bi-, and tri-specific engagers for antigen retargeting on cancer cells (31).

In the past, therapeutic monoclonal antibodies (mAbs) [e.g., rituximab (anti-CD20), cetuximab (anti-EGFR), lintuzumab (anti-CD33), and alemtuzumab (anti-CD52)] against the corresponding surface antigens on leukemia cells have positively contributed to the treatment but still lead to the development of resistance and an unsatisfactory response rate. Moreover, several high expressed antigens appear on non-transformed cells and, thus, therapeutic antibodies that recognize those target molecules may be scavenged and turned ineffective (32–37). Recently, with the advance in recombinant DNA technology, bispecific (CD16 × CD19 or CD16 × CD33) and trispecific killer engager (CD16 × CD19 × CD22) were developed to redirect NK cell cytotoxicity toward malignant cells, demonstrating significant increase of NK cell cytotoxicity and cytokine release against several CD19 expressing B cell lines. Miller et al. have shown that efficacy with CD16 × CD33 bispecific (BiKE) or IL-15-trispecific killer cell engagers (TriKE) successfully reversed CD33-positive myeloid-derived suppressor cells and stimulated NK cell-induced target cell lysis (38, 39).

Vyas et al. showed clearly that trispecific immunoligands (ULBP2-aCD19-aCD33 and ULBP2-aCD19-aCD19), designated as triplebodies (TBs), successfully retargeted short-time-activated (24 h) NK cells demonstrating increased NK cell-dependent killing activities of several target cells (MEC1, HL60, BV-173, and SEM) by using ULBP2 as a natural ligand to induce high expression levels of NKG2D receptors on activated NK cells. Moreover, activated NK cells in response to control TBs without ULPB2 domains showed a reduced IFNγ release and killing properties compared to full-constructed TBs (ULBP2-aCD19-aCD33) (40).

Based on our review from a clinical phase I/II study using IL-2 activated haploidentical NK cell for adaptive immunotherapy (Clin-Gov-No-NCT01386619) showing not only benefits but also limitations due to tumor immune escape mechanisms (TIEMs), we focused on those TBs in response to NK cells to overcome TIEMs (6, 41, 42). All experiments were performed to investigate specifically the efficacy of the employed ULBP2-aCD19-aCD33 against only CD19/CD33-expressing leukemia cells, which are mainly found in resistant antigen loss variants especially described as mixed lineage leukemia (MLL). In combination with primary donor NK cells, activated up to 14 days, we analyzed the TB-dependent improvement of retargeted recognition and cytotoxicity of those effector cells. In addition, possible side effects due to activated NK cells in the crosslink to these TBs should be evaluated.

## Materials and Methods

### Construction, Expression, and Purification of the Trispecific Immunoligand ULBP2-aCD19-aCD33

The ULBP2-aCD19-aCD33 TBs, kindly provided by Prof. Elke Pogge von Strandmann and Dr. Maulik Vyas, was constructed from immunoligands with high specificity for NKG2D receptors on NK cells and for CD19 and CD33 on AML cells that were efficiently expressed and secreted by HEK293T cells as previously reported (40).

### BV-173 Cell Line

The B cell precursor leukemia cell line BV-173 was purchased from Leibnitz Institute DSMZ (German Collection of Microorganisms and Cell Cultures) and maintained in RPMI-1640 Medium supplemented with 10–20% fetal calf serum (FCS). Cells were split every 3 days under cell culture conditions (37°C, 5% CO_2_). For functional assays, the cells were washed once with phosphate-buffered saline (PBS), centrifuged and adjusted to a final concentration of 2.5 × 10^5^/ml in TexMACS (Miltenyi Biotec) containing 5% human serum albumin (HSA).

### Toxicity Studies Containing T and B Cells

For toxicity experiments in response to TBs (ULBP2-aCD19-aCD33), T and B cells were isolated from fresh whole blood of healthy donors. The EasySep™ HLA Whole Blood B Cell and CD3 Positive Selection Kit (STEMCELL™ TECHNOLOGIES, Germany) was used to separate CD19^+^/CD20^+^ B or CD3^+^ T cells, respectively, by positive selection according to the manufacturer’s recommendations. The isolated cells were expanded in RPMI-1640 medium containing 10% FCS and in presence of a cytokine composition [final concentration: 50 IU/ml (IL-2), 100 IU/ml (IL-4), and 20 IU/ml (IL-10)]. The cells were split every 2–3 days under culture conditions (37°C, 5% CO_2_). These cells were washed once with PBS and adjusted to a final concentration of 2.5 × 10^5^/ml in TexMACS (Miltenyi Biotec) supplemented with 5% HSA. For toxicity assays, CD33 on T cells and CD19 surface expression on B cells were characterized by 10-color flow cytometry (FCM) analysis and then co-incubated with IL-2 activated NK cells (E/T ratio: 1:1) pretreated with 1 µg/ml TBs.

### Thawed Primary Human AML Cells

**Figure 1:**
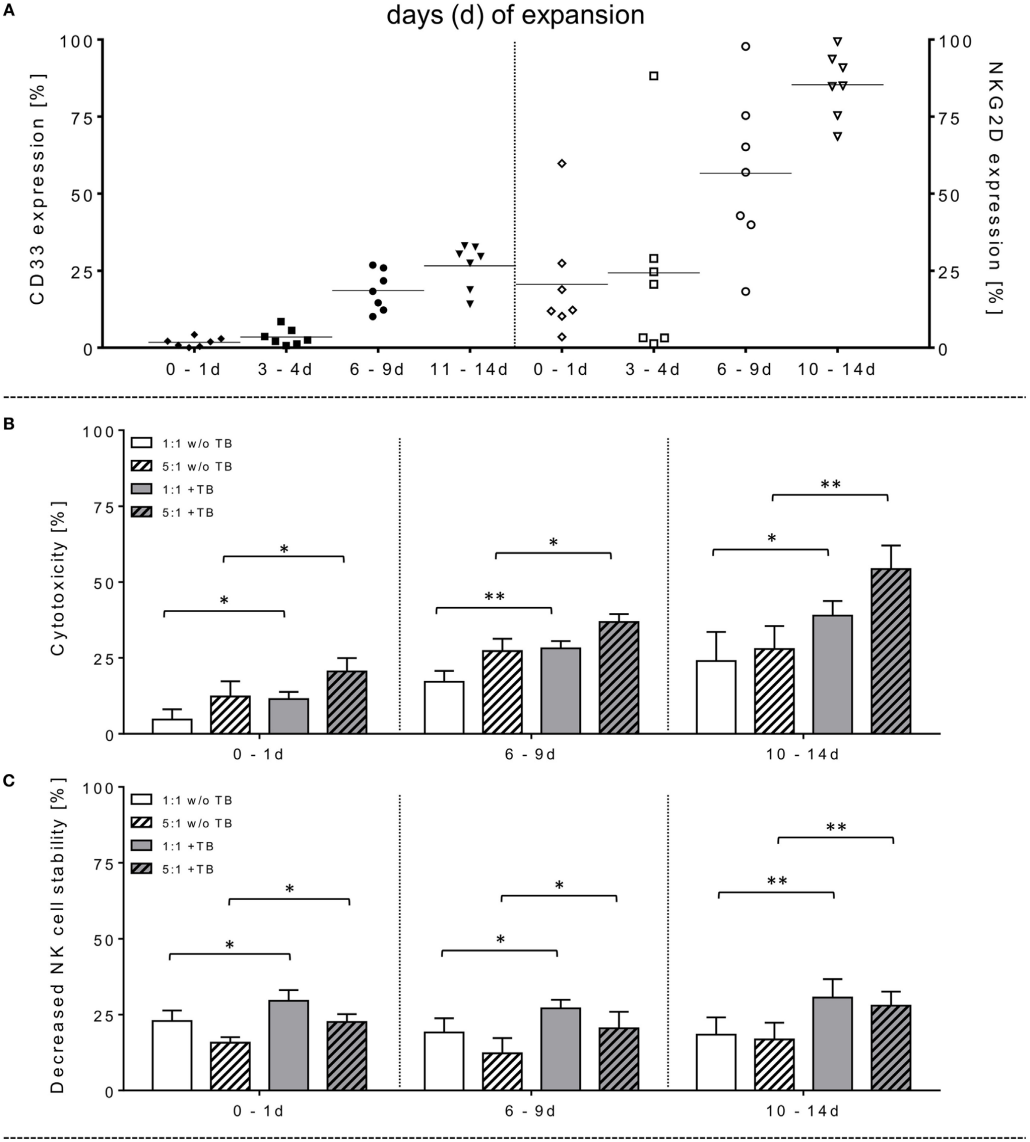

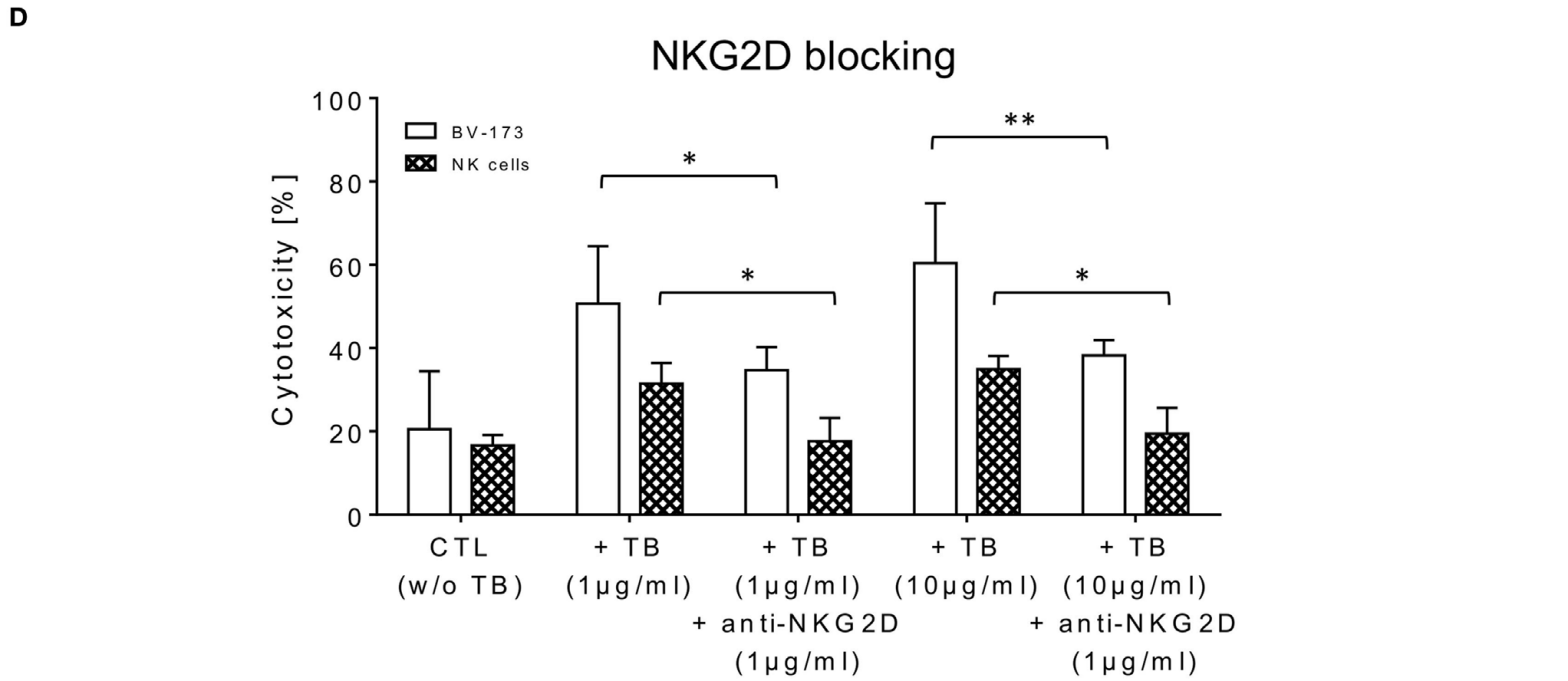
Phenotyping, effector cell stability and cytotoxic properties of activated natural killer (NK) cells alone or in combination with triplebodies (TBs) (ULBP2-aCD19-aCD33)-inducing effects against BV-173. **(A)** *In vitro* rearrangement of the NK cell phenotype was assayed immediately after effector cell separation as well as every 2–3 days within IL-2 expansion (1,000 IU/ml) for the following 14 days. In addition to monitoring of natural cytotoxicity receptors, co-expression of CD33, and natural killer group 2 member D (NKG2D) was also quantified for these unstimulated and expanded effector cells. **(B)** Effector cells were co-incubated for 4 h (37°C, 5% CO_2_, 250 rpm) with BV-173 cells at 1:1 or 5:1 E/T ratios, respectively, and killing activities (%) were determined in presence (+TB) and absence (w/o TB) of 1 µg/ml TBs (ULBP2-aCD19-aCD33) by flow cytometry. **(C)** Impact of 1 µg/ml TBs on the NK cell stability during effector-target-interactions corresponding to E/T samples. **(D)** To inhibit TB effects on NK cell killing and stability, receptor–ligand-(NKG2D-ULPB2)-binding to activated NK cells (expansion period: 10–14 days) were blocked by pre-incubation with anti-NKG2D monoclonal antibodies (1 µg/ml for 20 min) in presence of 1 or 10 µg/ml TBs, respectively. NKG2D-dependent killing rates (white columns) of those incubated NK cells in response to BV-173 cells and NK cells stability (columns with squared pattern) were analyzed after 4 h (“NKG2D blocking,” ratio: 5:1, 37°C, 5% CO_2_). Data of cytotoxic results are shown as mean ± SD from 4 to 6 experiments for each sample in duplicates. Statistically significant difference: *p ≤ 0.05 and **p ≤ 0.01.

Thawed primary AML samples from three different patients [French-American-British classification system: M0 or M5, respectively, kindly provided from Prof. M. Heuser, Hannover Medical School (MHH)] disclosing myeloid CD33 and B lymphoid CD19 surface marker expression were used as examples for antigen loss variants such as MLLs. These primary blasts were washed twice with PBS containing 10% FCS and treated with DNase I to avoid cell clumping. Cells were cultured up to 2 weeks in IMDM supplemented with 10% FCS, Penicillin/Streptavidin, l-Glutamine, and 20 ng/ml each of IL-3, IL-6, SCF, G-CSF, and GM-CSF. As an essential control for differentiation, marker expression, and stability, CD33, CD19 surface levels and cell viability of thawed AML samples were monitored every 2–3 days over a time period about 2 weeks (Figure 1A). Afterward, primary leukemic cells were washed with PBS and adjusted to 2.5 × 10^5^/ml in TexMACS (Miltenyi Biotec) containing 5% HSA and used for cytotoxicity assays in different E/T ratios in response to NK cells and TBs. Additional mono-cultured primary target cell controls were monitored during cytotoxicity assays under normal culture conditions (37°C, 5% CO_2_) and analyzed by FCM to estimate the viability, CD33 and CD19 surface expressions.

### Untouched Isolation and Expansion of Primary CD56^+^CD3^−^ NK Cells

Up to 30 ml anticoagulated whole blood from different healthy donors was used to separate “non-touched” primary human NK cells without density gradient centrifugation using MACSxpress^®^ NK Cell Isolation Kit (Miltenyi Biotec, Germany) according to the manufacturer’s recommendations. Based on the expansion protocol from the previous clinical phase I/II NK cell study (42), we improved the protocol and expanded these freshly isolated NK cells (purity: 97.8 ± 1.4%) in NK MACS^®^ basal medium (Miltenyi Biotec, Germany) containing 5% AB serum (human) and 1,000 IU/ml IL-2 up to 14 days (d) as described previously (43).

### Cytotoxicity Assay

To assess the NK cell-mediated killing activity in the presence and absence of TBs (ULBP2-aCD19-aCD33), we optimized a no-wash, single platform cytotoxic assay based on FCM (Navios, Beckman Coulter, Germany). This functional assay is based on the recovery of the viable effector and target cells after cytotoxic interaction within a predefined period of time (4 h). Initially, the surface expression of relevant antigens on cultured effector and target cells were determined by FCM as an essential control prior to each approach of this cytotoxicity assay. These phenotypic determinations included specific markers, such as CD45, CD56, CD16, CD33, and NKG2D (CD314) for NK cells and CD9, CD19, CD33, and HLA-DR for target leukemia cells. Further on, freshly isolated and cultured (0–14 days of expansion) NK cells were pre-incubated with various TB doses (TBs: 0.1–30 µg/ml). To determine TB-mediated cytotoxic effects against CD33^+^/CD19^+^ leukemia target cells, pre-coated or non-treated (control) NK cells were co-incubated in different ratios (E:T ratio: 1:1, 5:1) with the leukemic cell line BV-173 and/or primary leukemia blasts. To prevent insufficient stirring of incubated samples or cell sedimentations during cytotoxic cell contacts the co-cultured suspensions were shaken in an CO_2_-incubator (CO2 cell, 170-400 Plus, RS Biotech, Scotland) for up to 4 h (37°C, 5% CO_2_, 250 rpm). Afterward, effector cells were stained with mAbs by using CD45 KO (Krome Orange), CD56 PC-7 (Phycoerythrin-Cyanine-7) and CD16 APC (Allophycocyanin) in order to exclude the effector cells from leukemia cells stained with CD9 FITC (Fluorescein Isothiocyanate), CD34 PE (Phycoerythrin) and HLA-DR PB (Pacific Blue). Toxicity against effector and/or target cells with and without TBs was calculated as the increased loss of viable cells (43–46):

$$\text{Cytotoxicity}=\left(\begin{array}{l} \text{1}-{\text{concentration}}_{\left[\text{co-cultured target cells/μl}\right]}\\ {\text{over concentration}}_{\left[\text{target control cells/μl}\right]} \end{array}\right)\times \text{100\%}$$

To prove TB specificities, the primary NK cells were pre-incubated (20 min, 37°C, 5% CO_2_, 250 rpm) with 1 µg/ml anti-NKG2D to block the redirected cytotoxicity in response to TBs against leukemia blasts.

### CD107a-Degranulation Assay

Concurrent to our cytotoxicity assays, we assessed the NK cell degranulation by monitoring the cell surface expression of the lysosomal protein CD107a *via* FCM. NK cells were also co-incubated with leukemia cells at the same E:T ratios in response to TBs (ULBP2-aCD19-aCD33). Cells were stained with PE-conjugated anti-CD107a mAbs and incubated for 1 h at 37°C, 5% CO_2_. Phorbol 12-myristate 13-acetate and Ionomycin (I) (Cell stimulation cocktail from eBioscience) were used as a positive control whereas NK cells alone served as unstimulated baseline parameter. After stimulation, Monensin (1:1,000; eBiosciences) and GolgiPlug (1:1,000; BD Biosciences) were added to the samples. These batches were incubated for additional 3 h. Subsequently, cells were washed, stained and analyzed by FCM (see chapter: “Cytotoxicity Assay”).

### Cytokine Analysis

The multi-analyte flow assay kit (LEGENDPLEX™, BioLegend^®^, USA) was used for detection of soluble cytokines and pro-apoptotic markers, especially IFNγ, TNFα, perforin, GraA and GraB, and granulysin. Two sets of beads with known size and fluorescence allowed detections of those soluble molecules in supernatants that previously contained co-cultured effector and target cells. All analysis and evaluations were carried out according to manufacturer’s recommendations.

### Time-Lapse Microscopy

Redirected cell contacts and interactions between effector and target (E/T) or effector and effector (E/E) cells in presence of TBs could be monitored and followed by fluorescence scanning microscope (IX81, Olympus, USA). As a control before starting for those imaging experiments, surface expression levels of IL-2-expanded NK cells and cultured leukemia cells (BV-173) were characterized for CD3 (PB), CD9 (FITC), NKG2D (PE), CD33 (PE), CD56 (PC-7), CD16 (APC), CD19 (ECD), 7-AAD (PC-5.5), HLA-DR (PB), and CD45 (KO). Afterward, NK cells and BV-173 cells were intracellular stained with cell proliferation dyes (CFSE/eFluor^®^ 450, affymetrix eBiosience, USA). In the following, NK and BV-173 cells were co-incubated (E/T ratios: 5:1) on chamber slides over a time period of 8 h in response to 10 µg/ml TBs under culture conditions (37°C, 5% CO_2_). Beside time-lapse movie experiments to follow specific cell migrations and interactions by designed tracking protocols (time-lapse movie: see Figure S1 in Supplementary Material), it was also possible to evaluate all recorded images containing specific E/T- and E/E-cell contacts and cluster formations by quantitative analyses using the Olympus scanR automated image and data analysis (quantitative evaluations/gating strategy: see Figure S2 in Supplementary Material).

### Statistical Analyses

Statistical analysis has been performed using GraphPad Prism v6.02 (GraphPad Software, San Diego, CA, USA). Results of different cytotoxic experiments were compared by the paired Student’s *t*-test in order to assess the significance of the NK cell-mediated cytotoxicity incubated in absence and presence of TBs (ULBP2-aCD19-aCD33). Statistical evaluations of surface expression levels are indicated as median with range in the individual text parts. Differences were stated significant for a *p* ≤ 0.05 and *p* ≤ 0.01 (indicated as * and **, respectively). Minor differences were defined as statistically non-significant (n.s.). Unless otherwise declared, results of statistical evaluations from functional assays are indicated as mean ± SD and represent 4–6 independent experiments and measured in duplicates by FCM.

## Results

### TBs Increase Killing Activities of IL-2-Expanded NK Cells against Leukemia Cells

The capability of ULBP2-aCD19-aCD33 TBs to induce specific NK cell cytotoxicity against human leukemic cells was determined using the CD19- and CD33-double-positive BV-173 cell line with pre-B phenotype. Purified NK cells (97.8 ± 1.4% CD56^+^CD3^−^) were IL-2 activated and expanded for 14 days. The moderate-to-low expression levels of the NCRs on freshly isolated NK cells were markedly increased approximately 5.2-, 4.9-, and 1.4-fold for NKp30, NKp44, and NKp46, respectively (data not shown). Concomitantly, NKG2D revealed higher median expression levels on these expanded NK cells with 86.4% (range: 64.5–99.2%; 10–14 days) and 57.2% (range: 15.1–97.3%; 6–9 days) compared to unstimulated and early cultured NK cells (Figure 1A, right graph). Activated NK cell cytotoxicity rises with increasing duration of expansion time which correlates also with elevated NKG2D levels on these NK cells (Figure 1A). However, the NK cell-mediated cytotoxicity against BV-173 cells could be further enhanced at increased expansion periods by pre-incubation of NK cells with 1 µg/ml TBs (ULBP2-aCD19-aCD33) (Figure 1B). Accordingly, the NK cell killing activity in presence of TBs reached a maximum of cytotoxic average value of 33.2 ± 5.1% (E/T: 1:1) and 55.2 ± 8.8% (E/T: 5:1) with IL-2-cultured NK cells expanded for 10–14 days compared to significant lower cytotoxic levels of untreated NK cells [24.8 ± 9.4% (E/T: 1:1) and 26.8 ± 5.0% (E/T: 5:1)] (Figure 1B).

**Figure 2:**
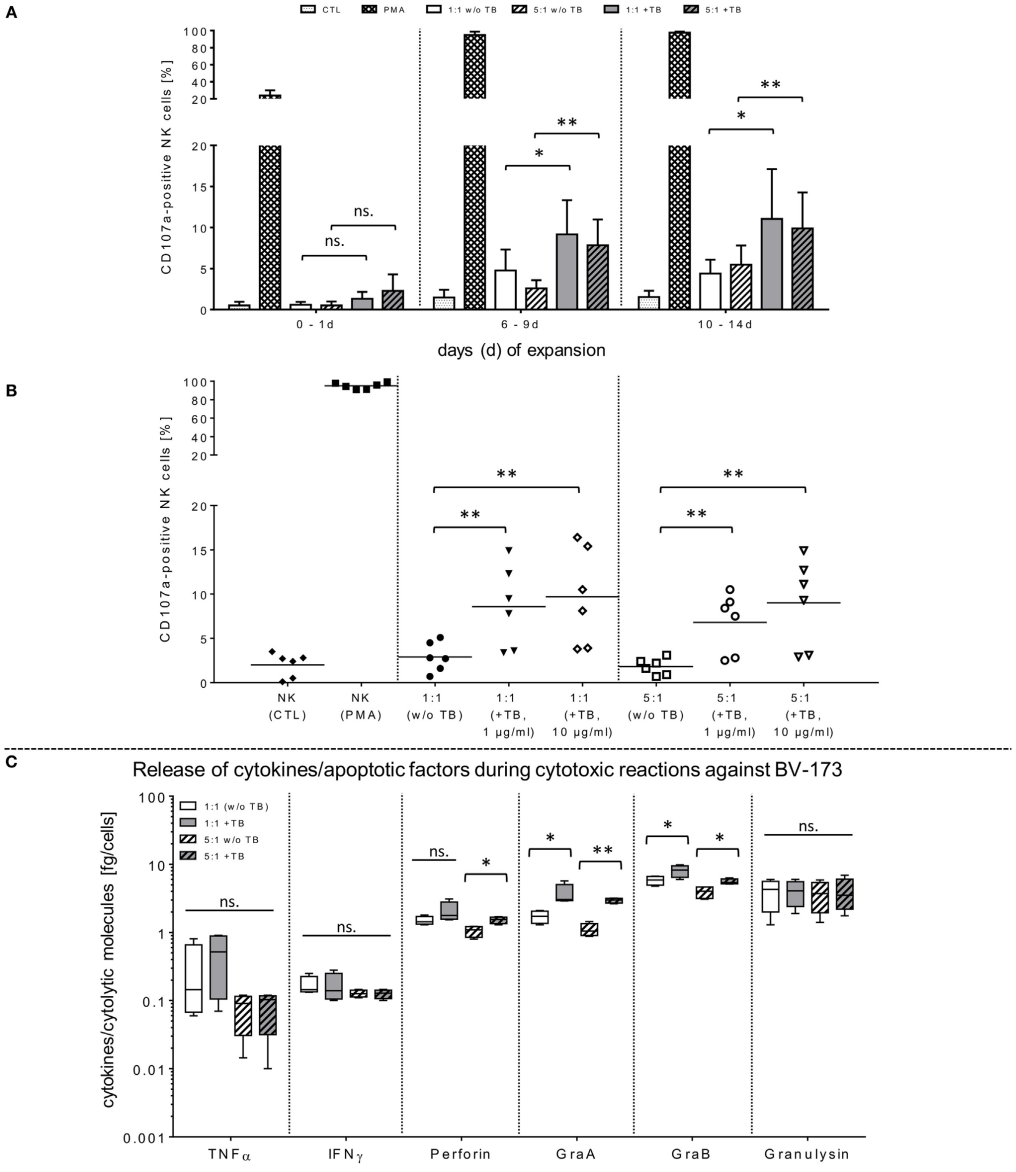

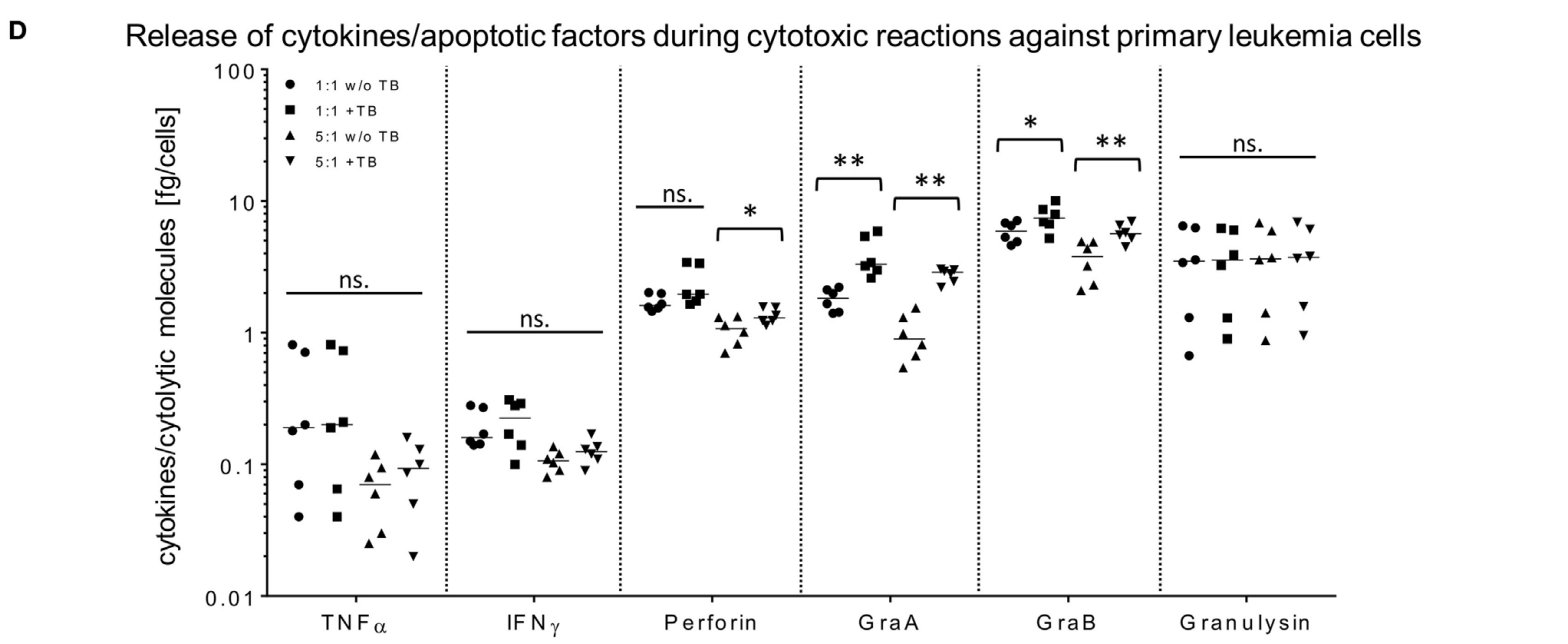
Functional activities of natural killer (NK) cells against BV-173 and primary leukemia cells in response to triplebodies (TBs). **(A,B)** Activated NK cells from latest expansion periods (10–14 days) were co-cultured with BV-173 cell line or with primary blasts from all acute myeloid leukemia patients, respectively. Afterward, the NK cell degranulation were detected by flow cytometry (FCM) analysis using the lysosomal protein CD107a in presence of 1 or 10 µg/ml TBs, respectively, under same experimental conditions as described in Figure 1. **(C,D)** Supernatants of different effector–target cell ratios against BV-173 **(C)** or primary patient blasts **(D)**, respectively, were collected after co-incubations over 4 h. Afterward, NK cell-mediated secretion of cytokines and pro-apoptotic markers were quantified by FCM at the indicated ratios in presence or absence of 1 µg/ml TBs. Data show mean ± SD from six experiments measured in duplicates. Statistically significant difference: *p ≤ 0.05 and **p ≤ 0.01.

In opposite to corresponding control TBs containing depleted ligand (ULPB2) or receptors (anti-CD33/anti-CD19), respectively, specific blocking antibodies were used to inhibit TB-induced cytotoxicity. For these NKG2D blocking experiments, different concentrations of TBs were used to show specific competitive inhibition by saturation of the target epitope with defined concentrations of blocking antibodies (anti-NKG2D) adjusted in several pre-experiments by titration of anti-NKG2D. Thus, inhibition of TB-dependent cytotoxicity by specific blocking of the receptor-ligand-(NKG2D-ULPB2)-binding sites could be achieved partially by pre-incubation of IL-2-expanded NK cells (10–14 days) with anti-NKG2D mAbs (1 µg/ml, 20 min) following treatment with 1 and 10 µg/ml TBs. This resulted in a reduction of cytotoxicity against BV-173 of 20.2-fold (1 µg/ml TB) or 25.8-fold (10 µg/ml TB) (Figure 1D). These results of cytotoxicity assays against BV-173 were largely consistent with the data from the degranulation assays. NK cells in different expansion periods were pre-incubated in absence or presence of 1 µg/ml TBs and co-cultured for 4 h (E/T ratios: 1:1, 5:1) to detect the lysosomal-associated membrane protein-1 (LAMP-1/CD107a) on NK cells as an mobilized cell surface marker following stimulation-induced granule exocytosis. TBs were able to elevate the degranulating subpopulation of NK cells in response to BV-173 cells at the indicated ratios with a maximum degranulation average of 11.1 ± 6.3% (E/T: 1:1) and 9.9 ± 4.6% (E/T: 5:1) on NK cells expanded for 10–14 days (Figure 2A). Interestingly, in some cases, non-significant lower degranulation of the effector cell at higher (5:1) compared to lower E/T ratios (1:1) in a TB-independent manner was observed (Figure 2A). Correspondingly, the analysis of cytokines and apoptotic markers released from TB-incubated NK cells during cytotoxic interaction against BV-173 cells showed increased concentrations for perforin, GraA and GraB, but no significant alterations of TNFα, IFNγ, or granulysin could be detected (Figure 2C).

### TBs Improve Killing Activity against Native AML Blasts by IL-2-Activated NK Cells

**Figure 3:**
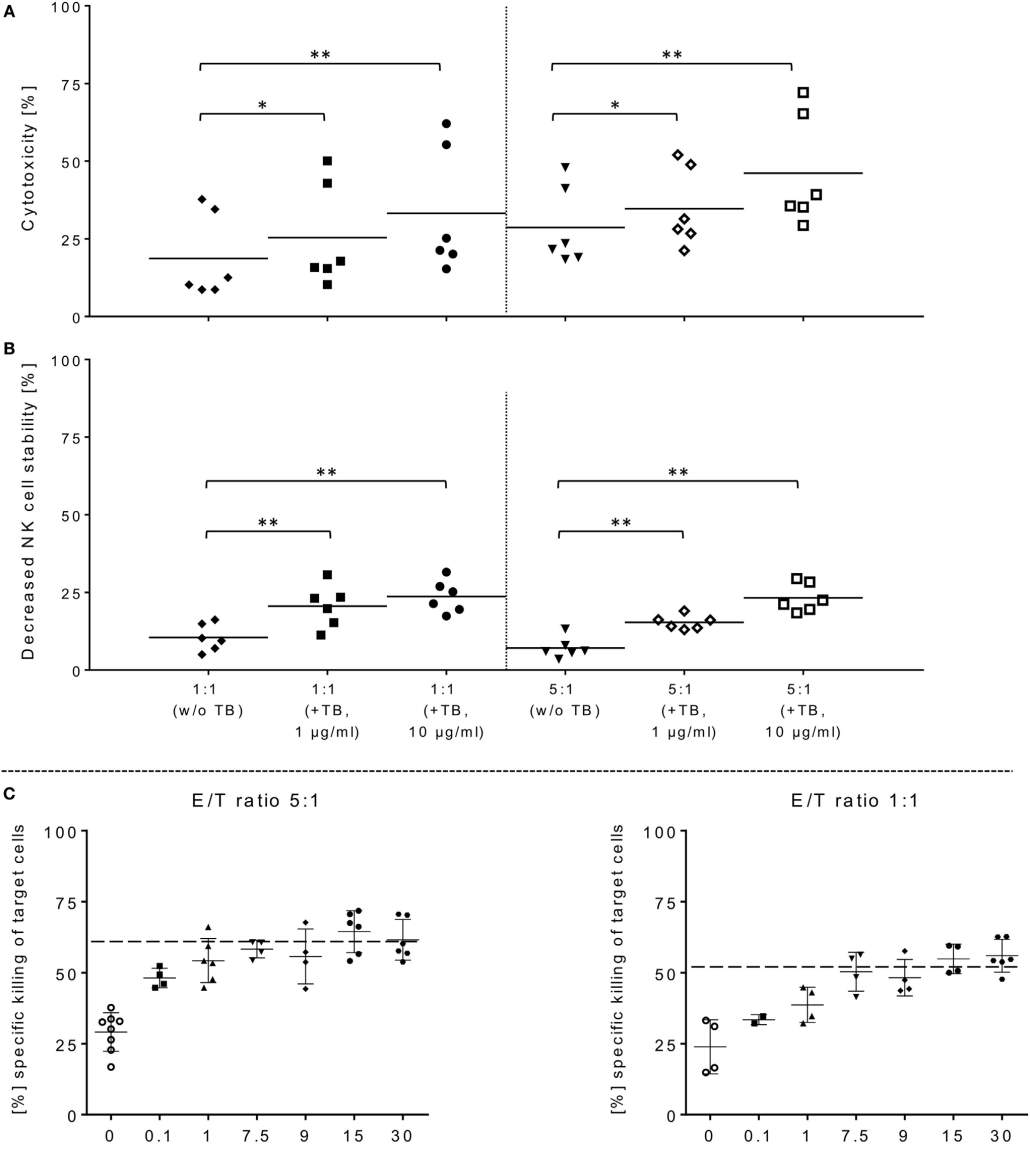

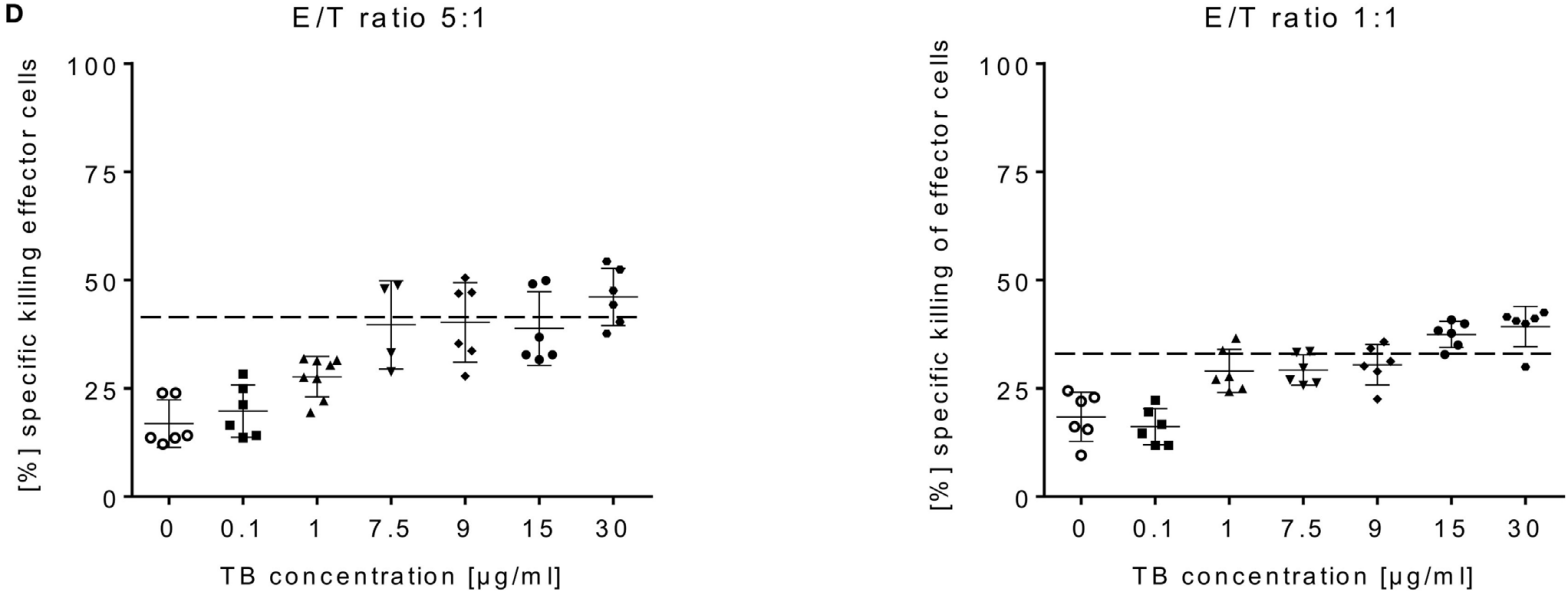
ULBP2-aCD19-aCD33-mediated natural killer (NK) cell killing of primary acute myeloid leukemia (AML) cells. **(A)** Activated NK cells were co-incubated with primary blasts from three different AML patients in presence of 1 or 10 µg/ml triplebodies (TBs), respectively, under same experimental conditions as described in Figure 1. **(B)** TBs-induced effects on the stability of NK cells were also determined corresponding to E/T samples (1:1, 5:1). **(C,D)** Estimate the maximum efficiency of ULBP2-aCD19-aCD33 against effector and target cells, different concentrations (0.1–30 µg/ml) of TBs were applied to activated NK cells (time period: 10–14 days) and BV-173 cells at the indicated ratios after 4 h (37°C, 5% CO_2_). Data present mean ± SD from six independent experiments measured in duplicates for each patient’s sample. Statistically significant difference: *p ≤ 0.05 and **p ≤ 0.01.

In order to demonstrate that TBs-(ULBP2-aCD19-aCD33)-treated NK cells also promote a cytotoxic effect against primary AML blasts, three different patient samples were thawed and NK cell cytotoxicity was assessed by FCM analyses. In the presence of TBs, the NK cell-mediated cytotoxicity against primary blasts from three AML patients was significantly enhanced compared to NK cell killing activities in absence of TBs. Moreover, the TB-mediated cytotoxic response was more pronounced at higher E/T ratios and TB concentrations. This resulted in improved killing activities of 1.4-fold (1.3-fold) with 1 µg/ml TBs and 1.8-fold (1.6-fold) with 10 µg/ml at E/T ratios of 1:1 or 5:1, respectively (Figure 3A). In accordance with our previous degranulation assays in response to BV-173 cells, increased CD107a-positive NK cell subsets could be identified during cytotoxic interaction at the indicated ratios with a maximum degranulation mean of 9.7 ± 5.2% (E/T: 1:1, 10 µg/ml TBs) and 8.9 ± 4.3% (E/T: 5:1, 10 µg/ml TBs) (Figure 2B). Cytokine and apoptotic marker detections in response to AML blasts revealed that pre-incubation of NK cells from expansion period 10–14 days with 1 µg/ml TBs resulted in elevated levels of perforin and GraA and GraB without changes in the amount of TNFα, IFNγ, and granulysin (Figure 2D). Corresponding to previous experiments in response to BV-173 cells, TNFα, and IFNγ showed a non-significant tendency to lower cytokine release at higher (5:1) compared to lower E/T ratios (1:1) which was also independent of the impact from TBs (Figure 2D).

### TBs Decrease NK Cell Viability during Cytotoxic Interactions against Leukemia Cells

Surface expression levels of CD33 were monitored within NK cell expansion over 14 days (Figure 1A, left graph). Beside increased NCRs and NKG2D, CD33 levels were also elevated on IL-2-activated NK cells with median expression levels of 27.9% (range: 15.8–33.8%; 10–14 days) and 21.6% (range: 11.0–28.2%; 6–9 days) compared to very low amounts in early expansion stages (Figure 1A, left graph). This led to the upcoming question whether the cytotoxic potential of the applied TBs analogous to the shown directed killing effects against AML blasts also has an unfavorable impact on the expanded NK cells themselves. Therefore, in addition to cytotoxic determinations against AML cells, the stability of the NK cells during cytotoxic interactions in the presence and absence of TBs was investigated in all cytotoxic experiments. Interestingly, in all E/T ratios pre-incubated with TBs, a marked decrease of these effector cells could be demonstrated both in response to BV-173 cells or AML blasts. Accordingly, TB-induced effector cell decrease reached a maximum average of 31.0 ± 6.4% (E/T: 1:1; 1 µg/ml TBs) co-cultivated with BV-173 cells (Figure 1C) or 24.9 ± 5.8% (E/T: 1:1, 10 µg/ml TBs) in response to primary blasts (Figure 3B), respectively. Interestingly, the NK cell viability seemed to be more reduced in 1:1 than in 5:1 E/T ratios only in presence of 1 µg/ml TBs (Figures 1C and 3B). By blocking of the receptor–ligand-(NKG2D-ULPB2)-binding (anti-NKG2D mAbs, 1 µg/ml, 20 min) in presence of TBs and BV-173 cells, this unfavorable effect on the stability of the NK cells could be almost completely abolished (Figure 1D).

### Dose Escalations of TBs (ULBP2-aCD19-aCD33) in Regard to Effector and Target Cell Stability

**Figure 4:**
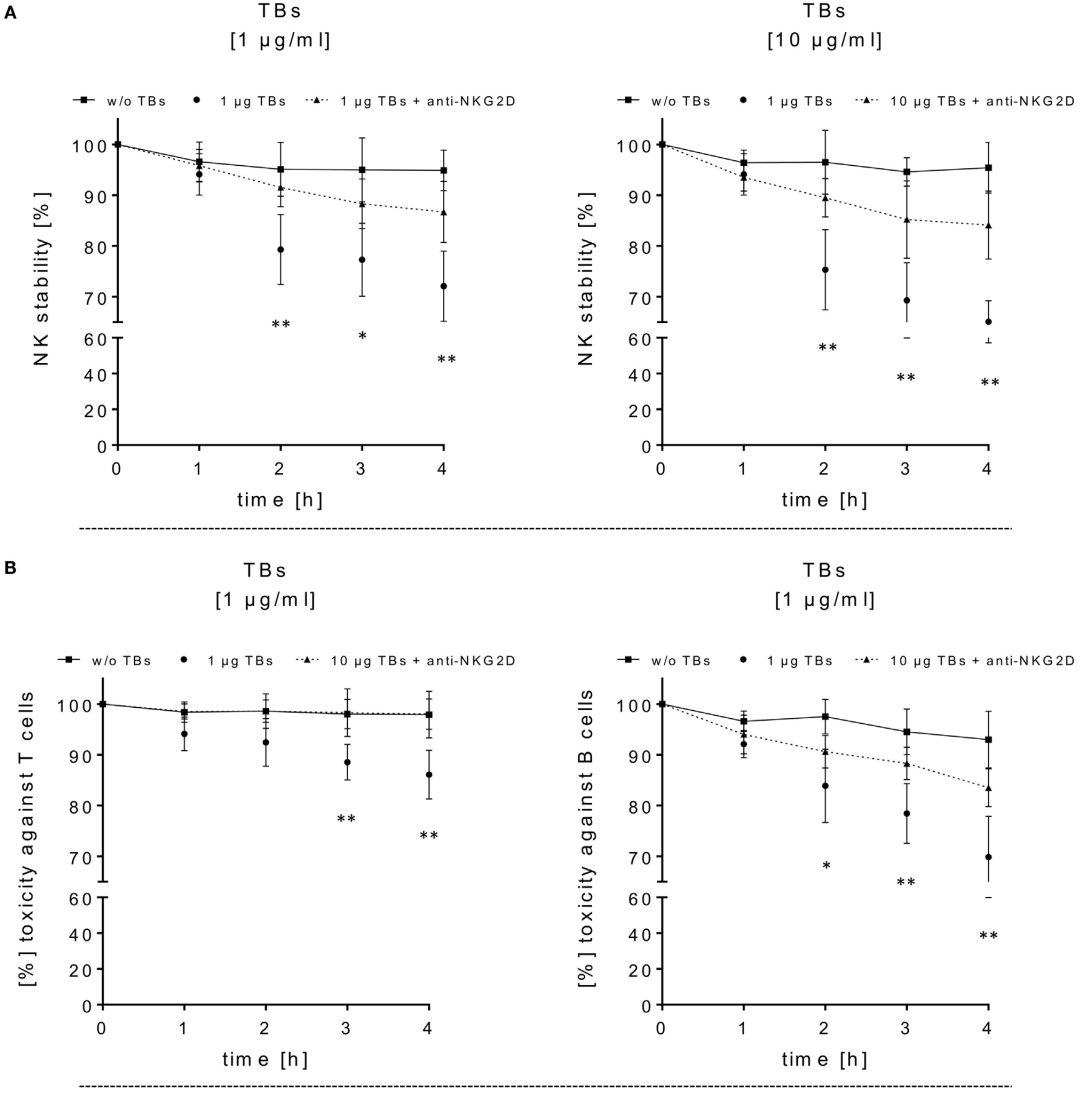

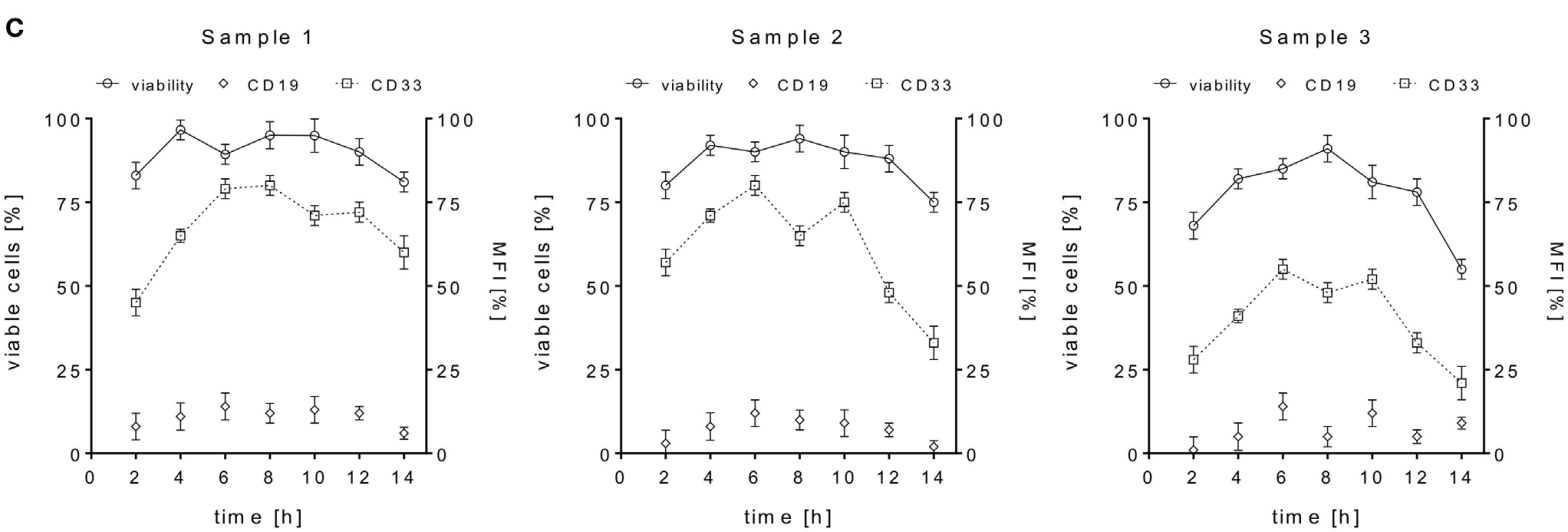
Adverse effects of ULBP2-aCD19-aCD33 against CD19- or CD33-positive lymphocytes. **(A)** Cell viability/stability experiments with activated natural killer (NK) cells alone in presence (dotted lines) or absence (black line) of 1 or 10 µg/ml triplebodies (TBs) (4 h), respectively, were performed for 4 h under culture conditions. To inhibit TB-induced toxicity effects, NK cells were pretreated with anti-natural killer group 2 member D monoclonal antibodies (mAbs) (1 µg/ml, 20 min; dashed line). **(B)** Co-incubation of activated NK cells and T or B cells (E/T ratios: 1:1) with (dotted line) or without (black line) 1 µg/ml TBs for 4 h (37°C, 5% CO_2_). TB-mediated effects could be blocked by pre-coated T and B cells with corresponding mAbs (1 µg/ml anti-CD33 or 1 µg/ml anti-CD19, respectively) for 20 min. Data present mean ± SD from 4 to 6 independent experiments measured in duplicates. Statistically significant difference: *p ≤ 0.05 and **p ≤ 0.01. **(C)** After thawing of three primary acute myeloid leukemia samples, the viability (○, left *y*-axis) and surface expression (mean fluorescence intensity) for CD33 (□, right *y*-axis) and CD19 (◊, right *y*-axis) were monitored every 2–3 days over a time period of 2 weeks as an internal control for the followed cytotoxicity assays.

In order to estimate in which concentrations those TBs (ULBP2-aCD19-aCD33) affect efficiently CD33^+^/CD19^+^ target cells and the stability of CD33^+^ effector cells, several dose-escalation experiments with highly activated NK (expansion period: 10–14 days) containing elevated CD33 levels in response to BV-173 cells were performed. Dose-escalation experiments of at least 0.1–30 µg/ml resulted in a maximum cytotoxic against target cells average of 60.2 ± 2.6% (52.4 ± 0.7%) from NK cells in response to BV-173 cells starting from a TBs concentration of at least ≥1 μg/ml and an E/T ratio of 5:1 or ≥7.5 μg/ml and an E/T ratio of 1:1 (Figure 3C). Moreover, a maximum toxicity mean response of 41.2 ± 1.5% (33.1 ± 0.8%) against co-cultured NK cells could be achieved from TBs concentrations of at least ≥7.5 μg/ml and an E/T ratio of 5:1 or ≥1 μg/ml at an E/T ratio of 1:1 (Figure 3D). Further toxicity experiments with activated NK cells and without any other target cells in presence of 1 or 10 µg/ml TBs (4 h), respectively, also demonstrated a pronounced decrease in the viability of the effector cells. Accordingly, a maximum reduction of 31.8% (24.0%) for effector cell stability after 4 h could be determined by 10 µg/ml (1 µg/ml) TBs (Figure 4A). However, pretreatment of NK cells with anti-NKG2D mAbs (1 µg/ml, 20 min) could partially neutralize the TB-mediated destabilization effects with a maximal blocking efficiency of 62.7% (64.0%) after 4 h in presence of 10 µg/ml (1 µg/ml) TBs (Figure 4A). These toxicity data raised the question whether these TBs, in addition to the demonstrated toxicity against activated NK cells, also revealed side effects against other lymphocytes, especially CD33^+^ T cells or CD19^+^ B cells. Therefore, we co-incubated (E/T ratios: 1:1) activated NK cells pretreated with 1 µg/ml TBs in response to T or B cells. This resulted in a moderate decrease of T cells, exhibiting only a weak CD33 surface expression, by approximately 12.1% after 4 h in contrast to higher reductions of 25.1% for B cells containing high CD19 expression levels (Figure 4B). However, pre-coating (20 min) of both lymphocyte subsets with the respective AK constructs (anti-CD33 or anti-CD19, respectively; each with 1 µg/ml) allowed blocking of the TB-induced toxicity (1 µg/ml TBs) down to 0.3% for T cells and 10.2% for B cells after 4 h co-incubations (Figure 4B).

### TBs (ULBP2-aCD19-aCD33) Promote Cell Cluster Formations between NK and Leukemia Cells

**Figure 5:**
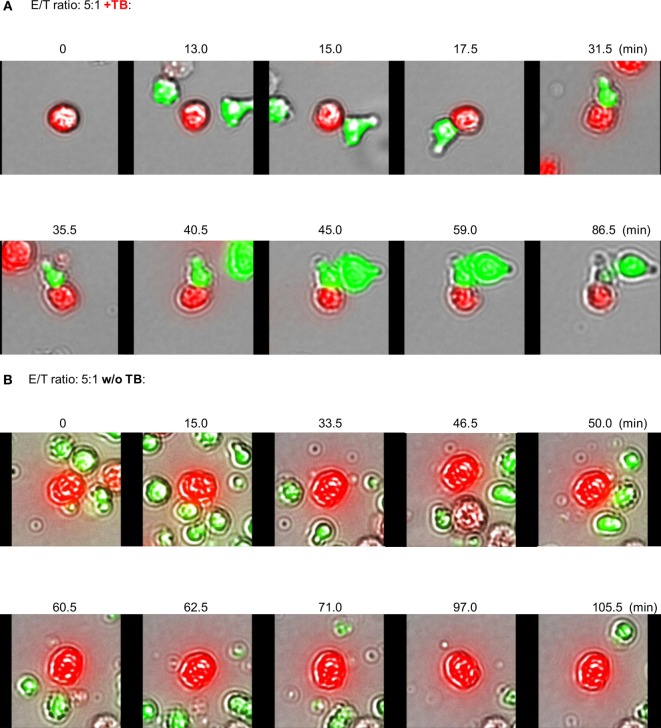
Redirected effector–target cell interactions in response to ULBP2-aCD19-aCD33. Activated natural killer (NK) cells (expansion period: 10–14 days) and BV-173 cells were labeled with CFSE (green NK cells) or eFluor^®^ 450 (red target cells), respectively, and co-cultured on chamber slides at the indicated E/T ratio for 8 h (37°C, 5% CO_2_) in presence **(A)** or absence **(B)** of 10 µg/ml triplebodies (TBs) (ULBP2-aCD19-aCD33) exemplarily shown for three different experiments. Specific Effector-to-target (E/T)- and Effector-to-Effector (E/E)-contacts were monitored by scanR analysis allowed the time-limiting tracking of cell migrations [0–86.5 min **(A)** and 0–105.5 min **(B)**] evaluated with a fluorescence scanning microscope (IX81, Olympus, USA), visualized in response to cell morphology and fluorescence.

Previous toxicity studies showed that our TBs also bind high-activated CD33^+^ NK cells resulted in reduced effector cell stability during the cytotoxic attack on leukemia cells. This led to the hypothesis that, in addition to increased cytotoxic contacts between NK and leukemia cells, elevated effector-to-effector cell contacts are responsible for higher effector cluster formations in the presence of TBs. This could partially neutralize the improved effect of TB-mediated NK cell killing activity. Therefore, CD33^+^ NK cells from late expansion periods (10–14 days) and BV-173 cells were intracellular stained with cell proliferation dyes (see Materials and Methods). Before fluorescent microscopy experiments were started, activated NK cells were also analyzed for NKG2D or CD33 (PE), respectively, CD56 (PC-7), CD16 (APC), CD3 (PB), and CD45 (KO) surface expression levels. Similarly, target cells were examined for following markers: CD9 (FITC), CD33 (PE), CD19 (ECD), 7-AAD (PC-5.5), HLA-DR (PB), and CD45 (KO). Subsequently, NK and BV-173 cells (E/T ratios: 5:1) were co-cultured over 8 h in presence of TBs (10 µg/ml) monitored by designed tracking protocols. Generated transmission and fluorescent images were quantitatively evaluated by described Olympus scanR acquisition analysis (quantitative evaluations/gating strategy: see Figure S2 in Supplementary Material). It was shown that specific E/T and also E/E cell contacts had increased significantly in presence to TBs (Figure 5A) compared to time-limited and unspecific/confused cell contacts in untreated controls (Figure 5B), exemplarily shown for two separated tracking runs (time-lapse movie: see Figure S1 in Supplementary Material). Accordingly, subsequent quantitative analyses confirmed the results of time-lapse monitoring by elevated numbers of E/T and/or E/E cell contacts in presence of TBs and resulted in higher cell cluster formations containing up to eight different effector and/or target cells shown in Tables 1 and 3. By contrast, only unspecific cell clumping and lower cell clustering containing smaller E/T or E/E cell numbers could be detected in absence of TBs (Tables 2 and 4) exemplarily presented for three independent experiments.

**Table 1:** Quantitative evaluations of E/T cell cluster formations.

Evaluation of E/T cell cluster formations (E/T ratio: 5:1 + TB)											

			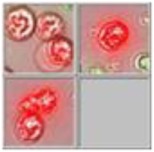	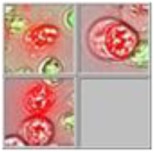	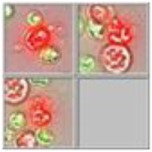	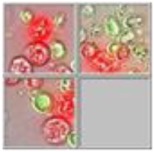	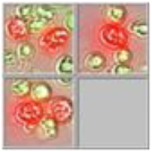	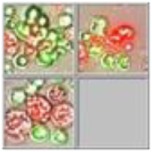	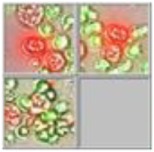	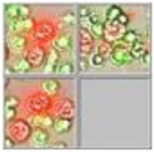	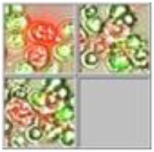

	Viable cells	Total cell clusters	T:T cell clusters	E:T cluster (1×)	E:T cluster (2×)	E:T cluster (3×)	E:T cluster (4×)	E:T cluster (5×)	E:T cluster (6×)	E:T cluster (7×)	E:T cluster (8×)
Gates	R01	R01/R02	R01/R02/R03	R01/R02/R04	R01/R02/R05	R01/R02/R06	R01/R02/R07	R01/R02/R08	R01/R02/R09	R01/R02/R10	R01/R02/R11
Cell umbers	8,157	3,334	650	679	1,003	715	132	95	41	16	3
(%)	100	40.9	8.0	8.3	12.3	8.8	1.6	1.2	0.5	0.2	0.04
	
						32.9% (2,684 E/T cell clusters)					

*Activated natural killer and BV-173 cells were co-cultured (E/T ratios: 5:1) on chamber slides over 8 h in presence of 10 µg/ml triplebodies (TBs) (37°C, 5% CO_2_). Specific cell interactions between effector and target (E/T) cells were analyzed by fluorescence scanning microscope (IX81, Olympus, USA)*.

**Table 2:** Quantitative analyses of E/T cell cluster formations in absence of triplebodies (TBs).

Evaluation of E/T cell cluster formations (E/T ratio: 5:1 w/o TB)											

			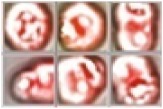	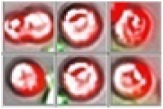	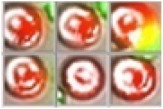	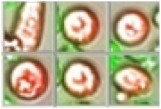					

	Viable cells	Total cell clusters	T:T cell Clusters	E:T cluster (1×)	E T cluster (2×)	E:T cluster (3×)	E:T cluster (4×)	E:T cluster (5×)	E:T cluster (6×)	E:T cluster (7×)	E:T cluster (S×)
Gates	R01	R01/R02	R01/R02/R03	R01/R02/R04	R01/R02/R05	R01/R02/R06	R01/R02/R07	R01/R02/R08	R01/R02/R09	R01/R02/R10	R01/R02/R11
Cell numbers	8,588	1,287	602	589	609	44	–	–	–	–	–
(%)	100	15.0	7.0	6.9	7.1	0.5	–	–	–	–	–
	
						14.5% (1,242 E/T cell clusters)					

*Natural killer and BV-173 cells were co-incubated (E/T ratios: 5:1) over 8 h (37°C, 5% CO_2_). Clustering effector and target cells were analyzed by fluorescence scanning microscope*.

**Table 3:** Quantitative analyses of E/E cell cluster formations.

Evaluation of E/E cell cluster formations (E/T ratio: 5:1 + TB)						

			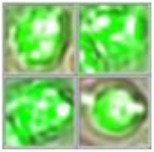	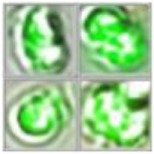	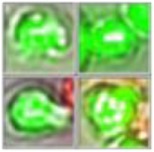	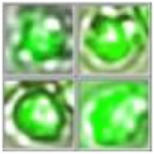

	Viable cells	Total E:E cell clusters	E:E cluster (1×)	E:E cluster (2×)	E:E cluster (3×)	E:E cluster (4×)
Gates	R01	R01/R02	R01/R02/R04	R01/R02/R05	R01/R02/R06	R01/R02/R07
Cell numbers	8,157	7,537	6,394	955	157	31
(%)	100	92.4	78.4	11.7	1.9	0.4

*Activated natural killer in response to BV-173 cells (E/T ratios: 5:1) were co-incubated over 8 h in presence of 10 µg/ml triplebodies (TBs) (37°C, 5% CO_2_). Specific effector-to-effector cell contacts were analyzed by fluorescence scanning microscope*.

**Table 4:** Quantitative evaluations of E/E cell clusters in absence of triplebodies (TBs).

Evaluation of E/E cell cluster formations (E/T ratio: 5:1 w/o TB)						

			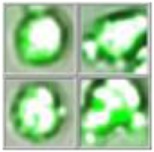	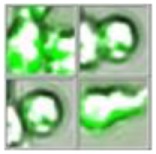	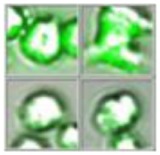	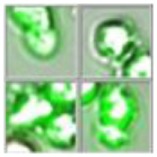

	Viable cells	Total E:E cell clusters	E:E cluster (1×)	E:E cluster (2×)	E:E cluster (3×)	E:E cluster (4×)
Gates	R01	R01/R02	R01/R02/R04	R01/R02/R05	R01/R02/R06	R01/R02/R07
Cell numbers	8,588	2,661	2,367	229	65	3
(%)	100	31.0	27.6	2.7	0.76	0.03

*Natural killer and BV-173 cells were co-cultured (E/T ratios: 5:1) over 8 h (37°C, 5% CO_2_). Clustering effector cells were analyzed by fluorescence scanning microscope*.

## Discussion

In our experiments, we could confirm the effectiveness of the (ULBP2-aCD19-aCD33) TBs in the crosslink with activated NK cells showing increased specific killing against a leukemia cell line (BV-173) and primary AML samples from three different patients compared to single use of NK cells only. Successful targeting was directed against both, the CD19 and CD33 antigen. The transmembrane glycoprotein CD19 (95 kDa) and the early myelopoietic antigen CD33 (approximately 67–75 kDa), seemed to be suitable and prominent surface markers to distinguish myelogenous leukemia cells from lymphoid or erythroid leukemia and were also clinically validated antigens for development of antibody-based immunotherapeutic (bi- or tri-specific) construct’s (47–49). However, it should be noted that human-activated NK cells also show a diversity of CD33 surface expression levels within different developmental stages (50–52).

Similar to our cytotoxic assays with the (ULBP2-aCD19-aCD33) TBs in response to leukemia cell lines and primary AML blasts, designed NKG2D-stimulating TBs that contained targeting against CD19 antigens (ULBP2-aCD19-aCD19) only displayed strong affinity to CD19 surface molecules on CLL cells. Vyas et al. (40) showed a significantly higher NK cell-mediated cytolytic activity in response to TBs (ULBP2-aCD19-aCD33 and ULBP2-aCD19-aCD19) against both, target cell lines (MEC1, BV-173, and SEM) and primary CLL blasts. The effects were independent from different E/T ratios (40). In our study as a proof-of-principle-experiment, we were able to inhibit the TB-induced cytotoxic specificity of IL-2-activated NK cells against BV-173 cells by blocking of NKG2D using anti-NKG2D mAbs. However, specific inhibition of these cytotoxic reactions could be achieved only partially and not fully by 1 µg/ml anti-NKG2D. This shows that in addition to the TB-induced killing activity, other cytotoxic mechanisms of activated NK cells are also present, which are NKG2D independent and could not be blocked in those analyses. In analogous experiments, Vyas et al. (40) achieved a decreasing specificity of TBs by pre-blocking the target antigens CD19 and CD33 on the surface of BV-173 cells. It was also shown that the NK cell-mediated cytotoxicity was strictly NKG2D dependent because control constructs lacking the ULBP2 domain could not induce IFNγ secretion and killing activity of co-incubated NK cells in response to leukemia cells (40). Further functional experiments revealed correlations of TBs-stimulated NK cell degranulations detected by increased CD107a^+^ effector cell populations. IFNγ secretion were also enhanced in presence of CD33-/CD19-expressing target cells that were inhibited by control constructs lacking the natural ligand ULBP2 for retargeting the NK cells *via* NKG2D receptors (40). By contrast, in our study, the functional assays showed no significant alterations for IFNγ or TNFα in collected supernatant samples after cytotoxic reactions. However, we detected TBs-dependent elevated NK secretion levels of apoptotic markers (perforin, GraA, and GraB) in response to both BV-173 cells and primary blasts. This correlated with an increased CD107a^+^ NK cell subset, but showed no alterations in the granulysin releases. This could be explained by the fact that probably the maximum time for intracellular productions of IFNγ and TNFα has long been exceeded by the long-term IL-2-driven NK cell expansion over 14 days. In contrast to our NK cell expansion protocol, Vyas et al., cultivated freshly purified NK cells only overnight (37°C, 5% CO_2_) in IMDM medium supplemented with 10% heat-inactivated FCS and human IL-2 (200 IU/ml) + IL-15 (10 ng/ml) (40). We concluded that this overnight cultivation in combination with both cytokines could induce an earlier IFNγ and TNFα secretion for these short-activated NK cells. Nevertheless, Vyas and our workgroup were able to show clearly improved cytotoxic properties of activated NK cells that were consistent with an optimized anti-leukemic efficiency. However, we detected a marked decrease in the stability of activated CD33^+^ NK cells during cytotoxic interactions against leukemia blasts confirmed by increased E/E cell contacts and higher effector cell clustering analyzed using fluorescence scanning microscope.

Future immunotherapy approaches containing primary NK cells in combination with examined TB-constructs should ensure that sufficient NK cell numbers and a strongly elevated NKG2D expression are available for an efficient receptor–ligand-(NKG2D-ULPB2)-binding as well as for complete eliminations of remaining leukemia cells, especially shown in high-risk patients. The significance of NKG2D could be also confirmed by several reports dealing with immunosurveillance and development of novel NK-based immunotherapies by using bispecific immunoligands targeting NKG2D receptors (7, 53–55). Concomitant experiments within our previous clinical phase I/II NK cell study (42) demonstrated that the NKG2D-dependent cytotoxicity against resistant neuroblastoma cells was strongly affected by immunosuppressing NKG2DLs, as one of multiple strategies to escape from immune-mediated eradication. This effect could be blocked by scavenging soluble NKG2DLs with IL-2-activated donor NK cells. As a result, NKG2D-dependent cytotoxic response was restored (41, 56). These results suggest that, in addition to a permanent characterization of NKG2D levels on NK cells, a closed monitoring of such critical immunosuppressive markers in patients’ plasma appears to be necessary before TBs are administered.

Since only controversial data concerning myeloid antigen CD33 (SIGLEC-3) expression on NK cells were published so far, this expression was also closely monitored concomitantly to NCRs/NKG2D characterizations. During 14 days of NK cell expansion, we could detect a transient higher CD33 surface expression level on late-expanded NK cells compared to unstimulated and early cultured primary NK cells. In accordance with these results, several subsets of NK cells were also found in human umbilical cord blood (CB) and in diverse distributions at different development stages in the peripheral blood (PB), lymph nodes, and spleen. Because of this distributions NK cell differentiations could occur at different anatomical locations (51). Interestingly, CD56^+^/CD33^+^ NK cell subpopulations identified in human umbilical CB revealed only a low cytotoxic effect against K562 target cells after IL-2-triggered expansion, whereas higher cytolytic effects were observed in response to activated CD56^+^/CD33^−^ NK cell subsets (50, 52).

Correspondingly to our experiments, increased CD33 expression levels were proven only in a subset of IL-2-cultured NK cells compared to ubiquitous elevations of NCRs and NKG2D surface expressions detected on all NK cells during 14 days of expansion. The viability of these CD33-expressing effector cells was adversely affected in presence of TBs (ULBP2-aCD19-aCD33) and resulted in a diminished cytotoxic response against leukemic blasts. In addition to the shown toxicity against CD33^+^ NK cell subsets, adverse effects toward the viability of T and B lymphocytes could also be observed, which were explained according to target antigen expressions (CD19 or CD33) on those lymphocytes. Besides the well-studied CD19 surface levels that are expressed during all development stages of B cells with the exception of differentiated plasma cells (57, 58), human T cells express also a low amount of CD33. Interestingly, both T and NK cells show similarly high surface expressions of activation markers (CD25, CD28, CD38, CD45RO, or CD95) (52). This could explain the observed toxicity of activated NK cells in the presence of TBs (ULBP2-aCD19-aCD33) against T and B lymphocytes. Therefore, a close-meshed patient monitoring and examination of PB-derived immune status from AML patients should be implemented.

## Conclusion

Our results indicate that TBs, especially ULBP2-aCD19-aCD33, are able to increase cytolytic properties of activated NK cells. This could be clearly demonstrated against both leukemic cell line BV-173 and primary AML blasts, but with some unfavorable toxicity effects against own effector cells and further adverse effects against T and B lymphocytes.

In summary, the experiences of our previous clinical phase I/II NK cell study for adaptive immunotherapy (Clin-Gov-No-NCT01386619) (6, 41, 42) and our results suggest that highly activated NK cells in combination with TBs, especially ULBP2-aCD19-aCD33, might be an innovative strategy for efficient redirected eliminations of resistant AML cell. Therefore, it is necessary to monitor both CD33 and NKG2D expression levels on *ex vivo* expanded NK cells and leukemic blasts isolated from PB of AML patients to improved therapeutic benefit.

## Ethics Statement

Pre-clinical development of an antibody-based triple body for NK cell-mediated immunotherapy of pediatric acute leukemia was approved and assessed by the Ethics Committee of Hannover Medical School (MHH) (ethical number: 2628-2015).

## Author Contributions

UK, ES, and SK designed the study, while AS, SK, and UK were mainly responsible for the performance of this study. More in detail, TG, NM, AS, and OO realized the experiments containing trispecific immunoligand ULBP2-aCD19-aCD33 (the so-called triplebodies [TBs]). SK, TG, NM, OO, and AS carried out the quality control analyses, including cell characterizations, effector cell killing activity and degranulation, time-lapse microscopy, and the enumeration of the cytokine secreting cells. MV and ES were responsible for construction, expression, and purification of the engineered trispecific immunoligand ULBP2-aCD19-aCD33. MH provided primary leukemia samples from different AML patients. SK wrote the manuscript, while UK, LA, and ES contributed to helpful discussions and the careful approval of the final manuscript. LA reworked as a specialist for native English the manuscript in word, sentence and grammar.

## Conflict of Interest Statement

The authors declare that the research was conducted in the absence of any commercial or financial relationships that could be construed as a potential conflict of interest.
