# Corrigendum: Triplebody Mediates Increased Anti-Leukemic Reactivity of IL-2 Activated Donor Natural Killer (NK) Cells and Impairs Viability of Their CD33-Expressing NK Subset

**DOI:** 10.3389/fimmu.2018.02326

**Published:** 2018-10-08

**Authors:** Stephan Kloess, Alessa Ede Valverde da Silva, Olaf Oberschmidt, Tanja Gardlowski, Nadine Matthies, Maulik Vyas, Lubomir Arseniev, Michael Heuser, Elke Pogge von Strandmann, Ulrike Köhl

**Affiliations:** ^1^Institute for Cellular Therapeutics, IFB-Tx, Hannover Medical School (MHH), Hannover, Germany; ^2^Department I of Internal Medicine, University Hospital of Cologne, Cologne, Germany; ^3^Department of Hematology, Hemostasis, Oncology, and Stem Cell Transplantation, Hannover Medical School (MHH), Hannover, Germany; ^4^Experimental Tumor Research, Center for Tumor Biology and Immunology, Philipps University Marburg, Marburg, Germany

**Keywords:** natural killer cells, natural killer group 2 member D, triplebodies, ULBP2, CD19, CD33, immunoligands, acute myeloid leukemia

In the original article, we neglected to include the following funding information:

This study was supported by a grant from the Deutsche Jose Carreras Leukämie-Stiftung to ES (DJCLS R 1408).

The authors apologize for this error and state that this does not change the scientific conclusions of the article in any way.

The original article has been updated.

## Conflict of interest statement

The authors declare that the research was conducted in the absence of any commercial or financial relationships that could be construed as a potential conflict of interest.

