# Supplementary figures and images for: Triplebody Mediates Increased Anti-Leukemic Reactivity of IL-2 Activated Donor Natural Killer (NK) Cells and Impairs Viability of Their CD33-Expressing NK Subset
Source: Front Immunol. 2017 Sep 8;8:1100. doi: 10.3389/fimmu.2017.01100 (PMC5596090; doi:10.3389/fimmu.2017.01100)

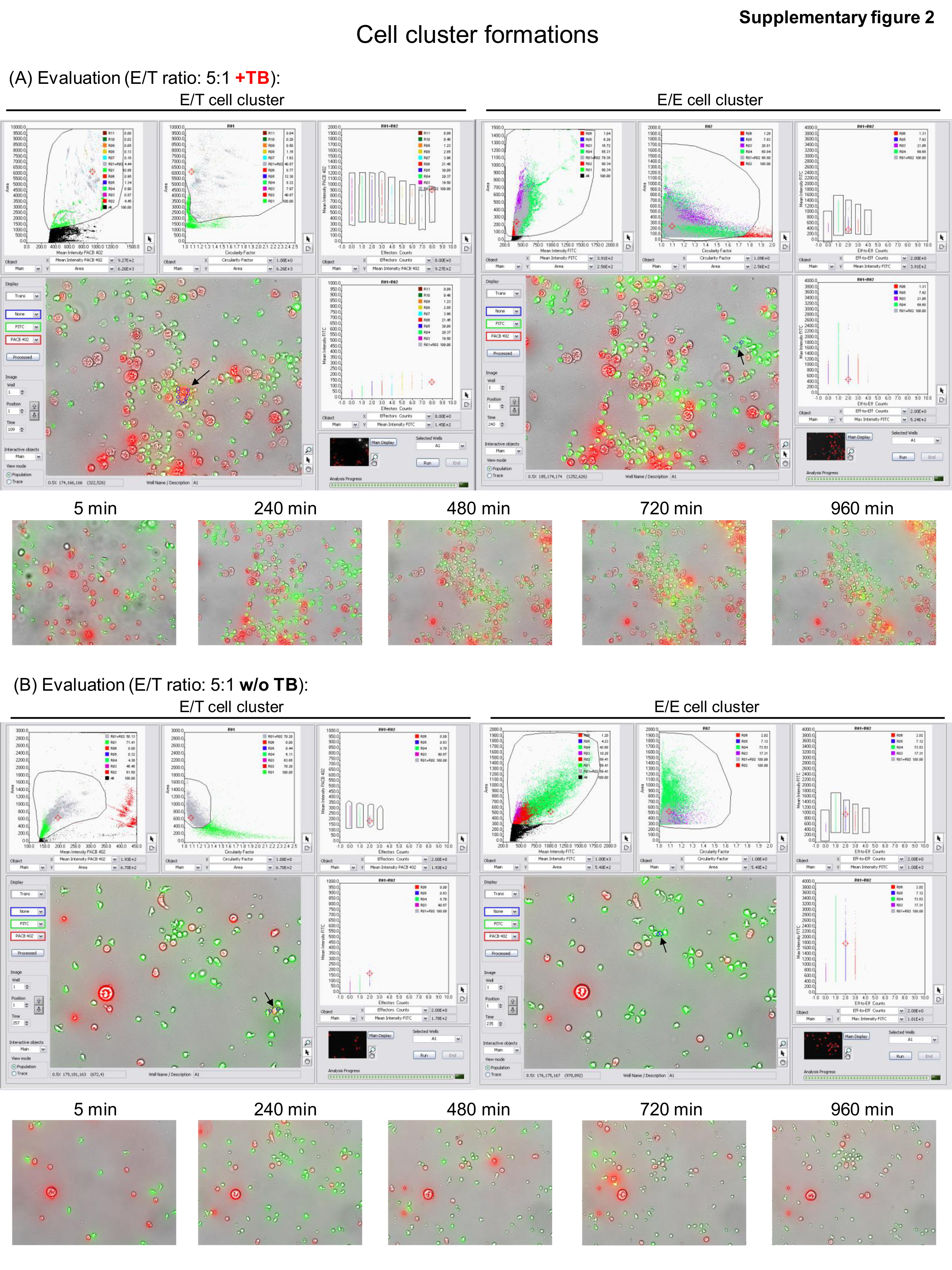

Supplement: Supplementary file 2 [file Image_2.TIF]
